# Energy Efficient Carbon Capture through Electrochemical pH Swing Regeneration of Amine Solution

**DOI:** 10.1021/acssuschemeng.3c08430

**Published:** 2024-04-30

**Authors:** Mu Lin, Clément Ehret, Hubertus V. M. Hamelers, Annemiek ter Heijne, Philipp Kuntke

**Affiliations:** † Wetsus, European Centre of Excellence for Sustainable Water Technology, P.O. Box 1113, 8900CC Leeuwarden, The Netherlands; ‡ Environmental Technology, Wageningen University, P.O. Box 17, 6700 AA Wageningen, The Netherlands

**Keywords:** carbon capture, electrochemical pH swing, amine solution regeneration, anion exchange membranes

## Abstract

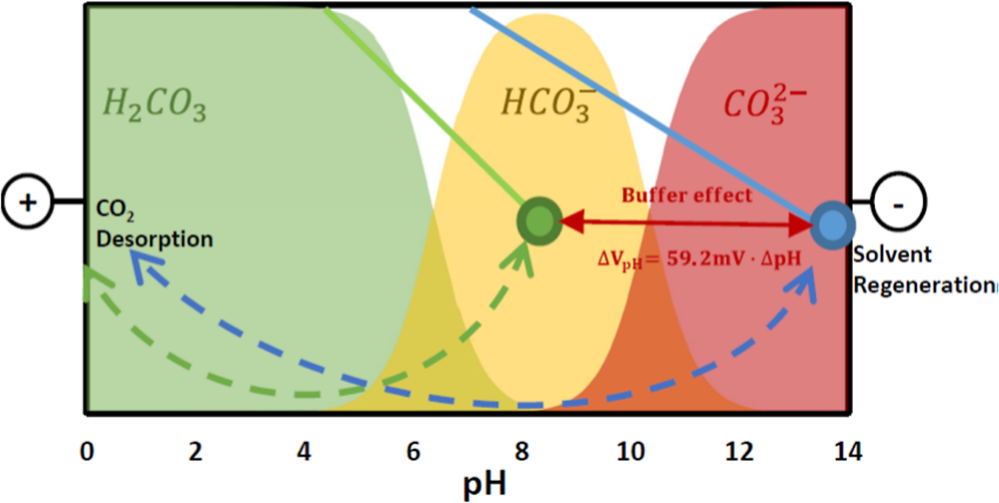

Carbon capture is widely acknowledged as a promising strategy for achieving negative emissions. Electrochemical carbon capture technologies are considered a viable alternative to conventional temperature swing processes. Among these, employing the hydrogen oxidation and hydrogen evolution reactions as a redox couple, along with an ion exchange membrane, offers an effective means of establishing a pH swing for desorbing CO_2_ and regenerating the alkaline solvent. However, the practical scalability of this approach is impeded by challenges such as high energy demands resulting from a high pH differential between anodic and cathodic environments and operation with solutions with a low conductivity, required to obtain an acceptable current yield. To address these limitations, this study introduces an innovative anion exchange membrane (AEM)-based electrochemical process for solvent regeneration. Our research demonstrates the advantageous utilization of amines as chemical buffers. Selecting an amine solution with a favorable p*K*_a_ (∼7 to 10) helps in maintaining bicarbonate as the predominant carbon species within the system, thereby ensuring a high current yield (>80%) across various operational conditions (current, load ratio, and solution concentration). Furthermore, our analysis indicates that the use of amine solutions effectively reduces the overpotential of the hydrogen evolution reaction due to a lower local pH. This results in a minimum energy requirement of 63 kJ/mol at a current density of 20 A/m^2^ to regenerate the solution (MDEA) while maintaining high (>99%) product (CO_2_) purity.

## Abstract 2

The buffering effect of amines allows for an energy-efficient solution regeneration in electrochemical systems employed for carbon capture.

## 1 Introduction

Climate change refers to long-term shifts in the temperatures and weather patterns. Human activities have induced climate change through rapid industrial growth over the past 60 years. Particularly, carbon dioxide (CO_2_) emissions contributed to such a shift.^1,2^ In the last 60 years, the atmospheric concentration of CO_2_ has increased by over 50% (from 280 to 420 ppm).^3,4^ A general roadmap to reduce greenhouse gas emissions has been depicted by the Intergovernmental Panel on Climate Change (IPCC) and the International Energy Agency (IEA) to limit the global temperature rise to less than 2 °C by the year 2060.^5^ This target could be reached by reducing the annual CO_2_ emission by 75% compared to the current annual emissions (35 GtCO_2_/year).^6^ For this goal, efforts in technology development have been made in various directions: renewables, nuclear energy, and carbon capture, utilization, and storage (CCUS).^7^

CCUS refers to a technology portfolio that combines capturing and concentrating of CO_2_ from diluted streams and processing (storage or conversion) of the enriched CO_2_. These sources can either be atmospheric air (0.04%)^8^ or more concentrated sources like flue gas (5–20%).^9^ Currently, the state of art in postcombustion CO_2_ capture technology is amine scrubbing with a thermal regeneration process. During this procedure, CO_2_ is absorbed in by a counterflow contactor operating at temperatures ranging from 40 to 70 °C. Subsequently, the solvent (e.g., ethanolamine) is heated to 100–150 °C and then purified with steam to yield pure CO_2_.^10^ The high energy demands for such a regeneration process (typically 2.7–3 GJ/ton CO_2_), which corresponds to approximately 20 to 30% of the power-generating capacity of a coal-powered plant, strongly hinder the proliferation of carbon capture facilities.^11^ Furthermore, the technology suffers from the potential degradation of the amine solvents under high-temperature regeneration.^12^ To overcome such challenges, there has been growing interest in developing electrochemical technologies that can reduce the energy consumption of carbon capture compared to that of this thermal amine carbon capture process. Electrochemical technologies allow for direct and efficient manipulation of CO_2_—solution equilibria under room temperature. Furthermore, electrochemical technologies are more flexible in adapting to renewable energy due to their simpler integration with, for example, solar or wind-derived electricity.^13,14^

In general, carbon capture is a two-step process, namely, absorption and desorption. During the absorption step, CO_2_ needs to be selectively separated from other gases usually with a bond formation. The subsequent desorption process releases CO_2_ by breaking or replacing these bonds. With electrochemical technologies, such a principle has been applied in studies on quinone derivatives,^4,15,16^ bipyridine,^17^ and electrochemically mediated amine regeneration (EMAR) methods.^6,18^ Another effective approach is to use protons as mediators in the absorption and desorption process. When absorption occurs, CO_2_ (H_2_CO_3_) donates its proton(s) to a proton acceptor such as an alkaline solution (generalized MOH_(aq)_), and bicarbonate (HCO_3_^–^) and/or carbonate (CO_3_^2–^) are formed (H_2_CO_3_ + *x*MOH ↔ *x*M^+^ + H_(2–*x*)_CO_3_^*x*–^, where *x* is either 1 or 2). By introducing protons into the system through an electrochemical oxidation reaction, HCO_3_^–^/CO_3_^2–^ can be released as CO_2_ from the solution *y*H^+^ + H_(2–*y*)_CO_3_^(2–*y*)–^ ↔ H_2_CO_3_, (where *y* is either 1 or 2). The pH difference of the solution directly or indirectly induced by the electrode reactions in processes is known as an electrochemical pH swing.^19^

**Figure 1:**
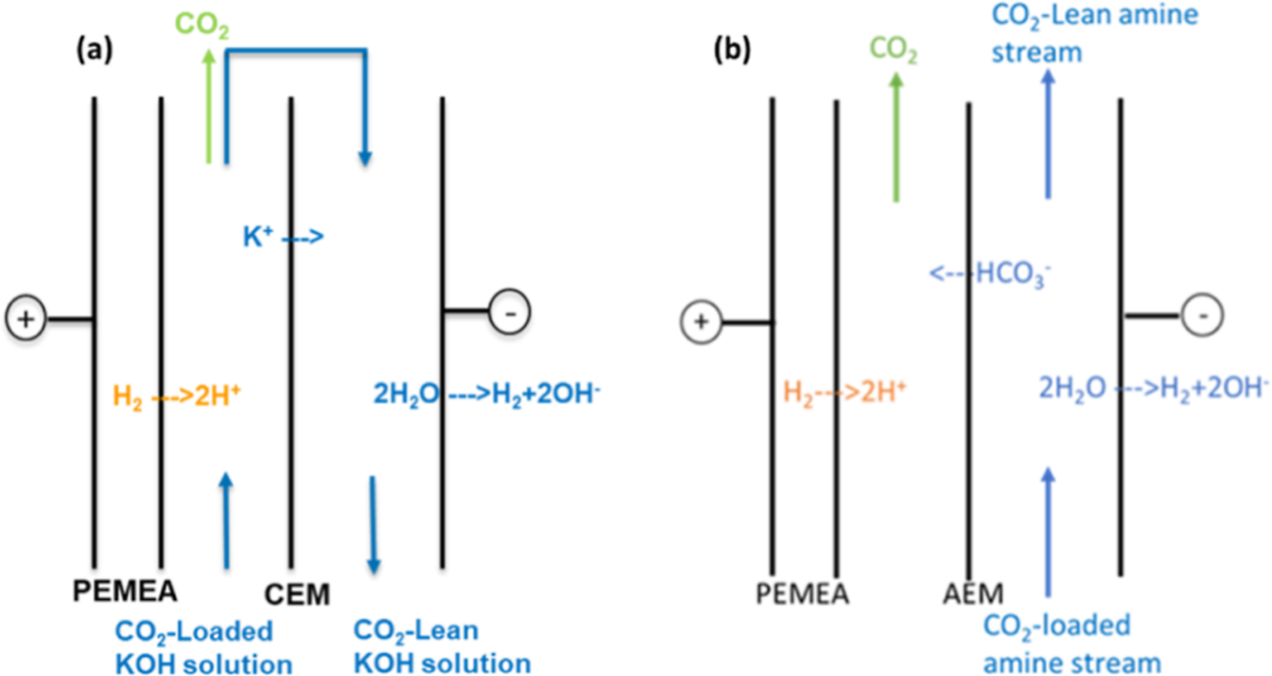
Spent alkaline solution regeneration by an electrochemical pH-swing process for carbon capture. (a) CO_2_ rich alkaline solution regeneration in a CEM design (based on work of Shu et al., 2020^20^), (b) CO_2_ rich amine solution regeneration in an AEM design (this study).

In general, the electrochemical pH swing solution regeneration process can benefit from prior knowledge obtained from (hydrogen) fuel cells (FCs) and water electrolyzers. A H_2_-recycling electrochemical system (HRES) has first been used for nitrogen recovery from wastewater.^19^ HRES is based on an anodic hydrogen oxidation reaction (HOR: H_2_ → 2H^+^ + 2e^–^) and a cathodic hydrogen evolution reaction (HER: 2H_2_O + 2e^–^ → 2OH^–^ + H_2_). Recently, a modified HRES has been demonstrated for the electrochemical pH swing carbon capture process using a cation exchange membrane (CEM).^20^ This system for direct air capture was used to regenerate spent NaOH solution, as shown in Figure 1a. The rich solution was regenerated by H^+^ ions released from the HOR in the acidifying compartment, leading to the release of CO_2_. Subsequently, the solution’s alkalinity was restored through OH^–^ ions from the HER in the cathode compartment, resulting in the production of a lean (alkaline) solution. The minimum theoretical voltage needed to establish an electrochemical pH swing is determined by the pH difference between the anode and cathode compartment considering a potential drop of 59.2 mV per pH unit (eq 1).^21^
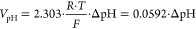
1where *R* is the ideal gas constant [8.314 J/(K mol)], *T* is the room temperature in Kelvin (297.15 K), *F* is the Faraday constant (96,485 C/mol), and 2.303 is the conversion factor from ln to log_10_. Our investigation has revealed that operating at a low load ratio results in a low current yield, as the introduced protons predominantly react with carbonate to form bicarbonate. Only at a specific load ratio threshold does the production of CO_2_ commence. Consequently, it becomes energetically inefficient to regenerate only a portion of CO_2_ from the solution. High recovery rate, however, requires a substantial pH shift: during extensive NaOH regeneration, the cathodic compartment experiences a highly alkaline pH (12–14). The introduction of the AEM system (Figure 1b) could resolve such a dilemma. In an AEM system, the current will transport the bicarbonate/carbonate to the acidifying compartment even at a low load ratio. The remaining of the unregenerated bicarbonate could possibly serve as the buffer to lower the local pH. Such an AEM system has recently been studied and demonstrated efficient by Muroyama et al.^22,23^ However, OH^–^ formed in the HER will complete with bicarbonate and carbonate ions for transport through the AEM, resulting in a reduction in the current yield. This is also reported by Muroyama et al.^23^ In theory, the utilization of amine-based solutions has the potential to address this issue. The presence of amines, typically characterized by p*K*_a_ values between 7 and 9, leads to a limitation in OH^–^ concentration due to the amine buffering effect. The reduction in OH^–^ concentration not only enhances the current yield but also impacts the HER redox potential, thereby lowering the overall applied cell voltage. This buffering effect has been extensively discussed within the fuel cell community over the past decade.^24−29^ In essence, the incorporation of a buffering agent offers advantages by lowering the local pH at the electrode and reducing the concentration overpotential.

In this context, we studied an AEM-based electrochemical regeneration system with amines as the solution. We show the proof of concept and insights into the reduction in energy input by applying an amine buffered system for the HRES pH swing solution generation process.

## 2 Experimental Section

### 2.1 Experimental Methodology

#### 2.1.1 Experimental Setup

As shown in Figure 1b, the regeneration setup was composed of three compartments: the cathodic compartment, the acidifying compartment, and the anodic compartment, which are separated by an anion exchange membrane (AEM) and a proton exchange membrane electrode assembly (PEMEA). A PEMEA consisting of a 15 × 15 cm^2^ Nafion 117 cation exchange membrane integrated with a 10 × 10 cm^2^ platinum-coated gas diffusion layer (0.5 mg_Pt_/cm^2^) (FuelCellEtc, TX) was applied as an anode, which separated the H_2_ stream from the acidifying compartment. The AEM separating the acidifying compartment from the cathodic compartment was separated by a Fujifilm type-I membrane (Tilburg, The Netherlands). A platinum-coated mesh electrode (2.5 mg/cm^2^, 10 × 10 cm^2^, Magneto Special Anodes BV, The Netherlands) was applied as the cathode. An identically sized Ru/Ir-coated (0.5 mg/cm^2^) titanium mesh electrode was applied as the current collector for the anode (Magneto Special Anodes BV, The Netherlands).

At the anode, H_2_ entered the gas diffusion layer of PEMEA, and HOR introduced protons in the acidifying compartment. At the cathode, the HER regenerated the alkalinity of the solution. In theory, for every mole of H_2_ consumed in the anode, 1 mol of H_2_ is generated in the cathode, resulting in a net zero production of H_2_ in theory. For practical reasons, H_2_ was fed to the anodic compartment from a gas cylinder (99.99%, AIR PRODUCTS, The Netherlands) externally, and the H_2_ generated from the cathode was not recycled.

Protons produced at the anode are transported through the PEM to the acidifying compartment, where they combine with bicarbonate/carbonate transported through the AEM to form CO_2_. In the outflow of the acidifying compartment, a membrane contactor (Liqui-Cel EXF model 2.5 in. × 8 in., 3M, Germany) was applied for the gas–liquid (CO_2_-solution) separation. The CO_2_ gas produced was first passed through a Nafion tubing (TUB-0003, CO2Meter.com, FL, USA) to remove the water vapor from the samples. Finally, gas production was measured using a mass flow meter (EL-FLOW Prestige FG-111B, Bronkhorst, The Netherlands), which measures the gas mass flow and displays results in normalized flow rate (mLn/min). Gas purity was assessed using an infrared CO_2_ sensor (SmartGAS Flow EVO, Germany) and validated by a micro gas chromatograph (Varian CP-4900, Agilent, CA, USA). Two peristaltic pumps (Masterflex L/S, Metrohm, The Netherlands) were used for solution recirculation in the experimental setup: one for the acidifying compartment and one for the cathodic compartment. Both pumps were set to a flow rate of 330 mL/min (∼10 cm/s).

The anode (PEMEA) potential and cathode potential were measured versus two Ag/AgCl reference electrodes (+0.23 V vs SHE) (ProSense B.V., The Netherlands). The pH of the solution was measured using a pH sensor (digiLine, JUMO GmbH and Co, Fulda, Germany). Galvanostatic control was carried out by using a potentiostat (Vertex.10A, IVIUM, The Netherlands). Two conductivity sensors (JUMO digiLine Ci HT10, Germany) were used to measure the conductivity. All sensors were placed at the exit of the electrochemical cell in the acidifying and cathodic compartment. The amine concentration in the solution was measured by ion chromatography (761 Compact IC, Metrohm, Switzerland).

#### 2.1.2 Experimental Procedure

Three different amines, ethanolamine (MEA, ≥99% purity), diethanolamine (DEA, ≥98% purity), and methyl diethanolamine (MDEA, ≥99% purity), were investigated in this study. These three amines are widely applied in the postcombustion carbon capture practice and also represent the different amine classes: primary, secondary, and tertiary amines, respectively. All chemicals used in this research were purchased from Sigma-Aldrich and used without further purification.

#### 2.1.3 Current Yield and Ohmic Resistance

**Figure 2:**
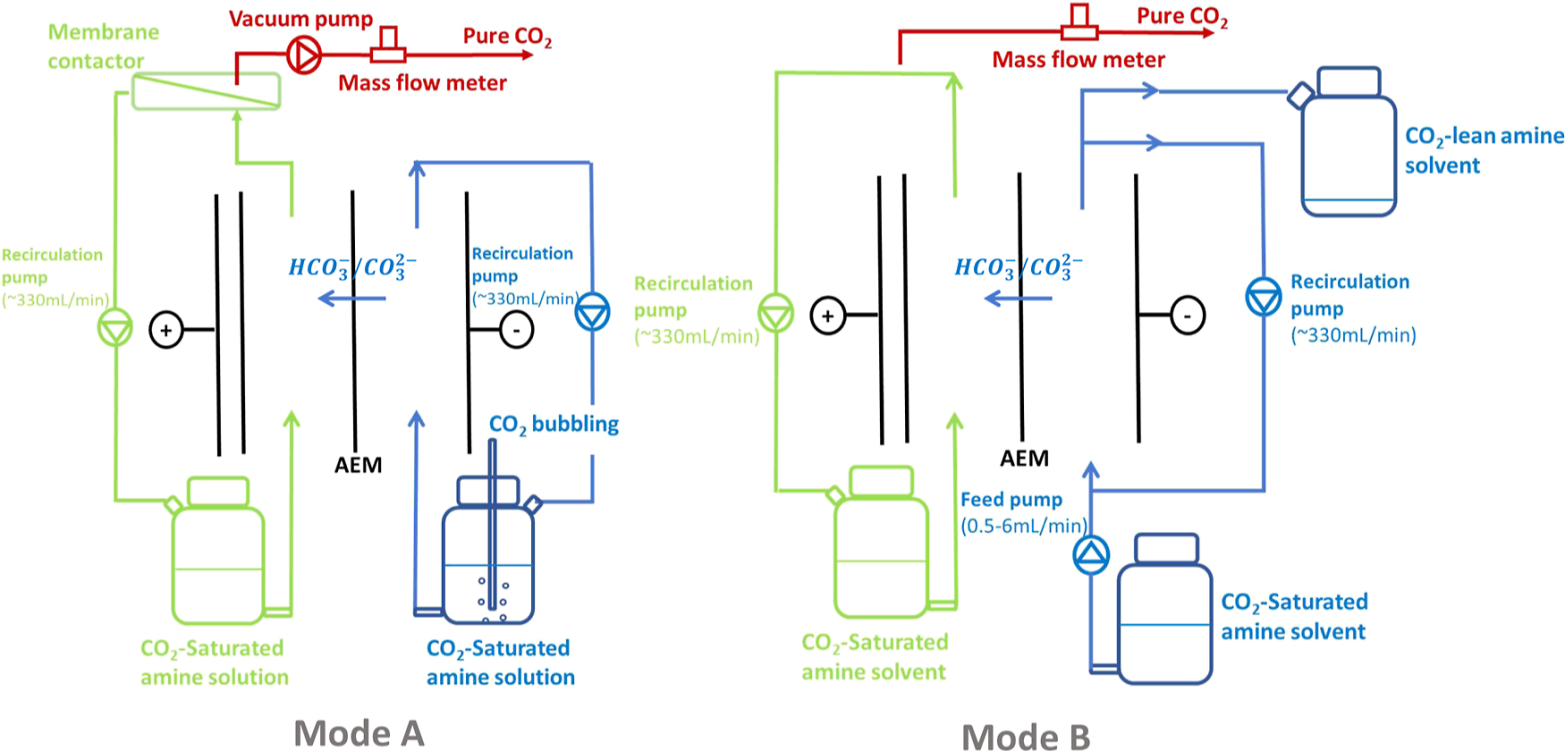
Illustrations of two different operation modes. Mode A: idealized process with constant saturated solution stream with vacuum pump (0.1 bar) and membrane contactor; mode B: continuous process with different load ratio scenarios without a vacuum pump and membrane contactor. In both modes, CO_2_ is transported as the form of carbonate/bicarbonate from the cathodic compartment (blue) to the acidifying compartment (green) and released as CO_2_ gas stream (red).

Figure 2 depicts the two different operation modes designed to understand the system performance. Experiments (mode A, Figure 2) were carried out to understand the current yield and ohmic resistance across different concentrations of amine solutions (0.5, 1, 1.5, and 2 M) under different current densities (0, 20, 40, 60, 80, and 100 A/m^2^). The current yield (*r*_*i*_) is described as the ratio between the equilibrium current corresponding to the amount of CO_2_ production versus the actual current applied as eq 2.
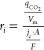
2where *j*_c_ is the applied current density (A/m^2^), *q*_CO_2__ is the CO_2_ production rate (L/s), *V*_m_ is the normalized molar gas volume (22.4 L/mol), and *A* is the active AEM membrane area (0.01 m^2^). The *r*_*i*_ is a measurement of overall current efficiency.

Each experiment at a given current density took ∼6 h to ensure steady state was established. Blank experiments with the same concentration (current density 0 A/m^2^) were carried out and the CO_2_ production from the blank experiments was subtracted from the corresponding experimental groups. This production of CO_2_ was due to the diffusion transport of CO_2_ (H_2_CO_3_) through the AEM from the concentration differences: in the cathodic compartment, the solution was in equilibrium with pure CO_2_, whereas in the acidifying compartment, the solution was in equilibrium with ambient air.

To provide a realistic energy consumption assessment, we examined the system with a set of experiments (mode B, Figure 2) that were performed by fixing the current density and adjusting the CO_2_ rich amine solution flow rate. In this case, we actively controlled the expected lean loading of solution in the effluent, i.e., create different scenarios for the extent of solution regeneration. Arguably these experiments mimicked operational scenarios which recovered CO_2_ from a 100% purity CO_2_ input. However, it was noteworthy that in a continuous process, the energy consumption was only subjective to the carbon loading of the effluent and not of the influent. In operation mode B, we also removed the vacuum pump and the membrane contactor. CO_2_ was stripped under the atmospheric pressure.

Here, we introduced the cation load ratio (*L*_c_) to better describe operation mode B. *L*_c_ was a factor defined by the ratio of current density and the theoretical equilibrium current density required to remove all the carbonate and bicarbonate ions
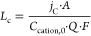
3where *j*_C_ is the applied current density (A/m^2^), *A* is the active AEM membrane area (0.01 m^2^), the *C*_cation,0_ is the molar concentration of cation (deprotonated MDEA or potassium K^+^ in this study) in the influent solution (mol/m^3^), *Q* is the flow rate of the influent solution (m^3^/s), and *F* is the Faraday constant (96,485 C/mol). When *L*_c_ = 1, it implies the supplied current can transport all carbonate or bicarbonate ions introduced in the system. When *L*_c_ < 1, it implies the supplied current was not sufficient to transport all the carbonate or bicarbonate ions introduced in the system. Compared with the experiments of mode A, CO_2_ was not continuously supplied to the amine solution. In both modes, energy consumption (E.C.) is defined and calculated according to eq 4.
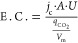
4where *j*_C_ is the applied current density (A/m^2^), *A* is the active AEM membrane area (0.01 m^2^), *U* represents the measured overall voltage (V) without *iR* compensation, *q*_CO_2__ is the CO_2_ production rate (L/s), and *V*_m_ is the normalized molar gas volume (22.4 L/mol).

In general, operation Mode A provides us an ideal operational condition for the electrochemical cell performance where the concentration of the studied rich solution remains the same in the influent, the cathodic compartment, and in the effluent. Operation mode B represents scenarios where the carbonic concentrations in the amine solutions were identical in the cathodic compartment and in the effluent, while different from those in the influent, and the concentration difference depends on the load ratio. Therefore, mode B represents a more realistic application scenario.

### 2.2 Polarization Curve Modeling for Cathode

To understand the effect of an amine buffer on overpotential for the HER, five different CO_2_-saturated solutions (1 M MDEA, DEA, MEA, KHCO_3_, and 0.5 M K_2_CO_3_) and KOH solutions were tested in mode A. In each experiment, the aforementioned identical solutions were recirculated in both the acidifying compartment and the cathodic compartment. To ensure equal (across the AEM) and constant concentrations of bicarbonate and carbonate species, CO_2_ was continuously supplied to the cathodic tank during the whole experiment for the groups of MDEA, DEA, MEA, and KHCO_3_ to ensure the saturation state of the solutions. For K_2_CO_3_, the solution recirculated in the cathodic compartment was replenished every 2 h during the experiment.

The current density was stepwise increased from 0 to 75 A/m^2^, and the potential difference between the reference electrode and the cathode was recorded. Each step was maintained for 1 h to reach equilibrium, and the potential was measured every 5 s. The average of the potential measurements of the last 30 min was used to calculate the cathode potential.

A simple model was applied to describe the electrode potential measured in this study to understand the effect of the amine buffer. In this model, the electrode potential measured consists of the equilibrium potential (*E*_eq_), the activation overpotential (η_act_), the ohmic overpotential (η_ohm_), and the contribution of mass transfer limitations. Due to the low current density applied (*j* ≤ 75 A/m^2^), mild concentration (1 M), and higher linear flow velocities (∼10 cm/s) in this study, mass transfer limitations were excluded from the model. Therefore, the cathode potential (*V*_cathode_) was described according to eq 5.^30^
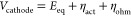
5

The equilibrium potential is defined by Nernst Equation (eq 6)
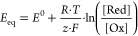
6where *z* is the number of electrons transferred in the redox reaction. After the species of the redox reaction (HER) is substituted in the Nernst equation (eq 6), the equilibrium can be rewritten as eq 7
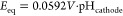
7

The activation energy is represented with the Tafel equation (eq 8)^31^
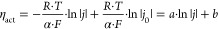
8where α is the charge transfer coefficient, *j* is the current density (A/m^2^), *j*_0_ is the exchange current density (A/m^2^), *a* represents the Tafel slope. With the assumption of a fixed electric resistance (*R*_Ohmic_) within the current density range in this study, the ohmic overpotential (η_Ohm_) is represented by a linear equation in eq 9
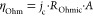
9where *j*_C_ is the current density, and *A* is the active cross-section area m^2^.

With the voltage–current (*U*–*I*) response measurement, the model was completed with the following steps. First, we used the data of the response from the higher current density (20–75 A/m^2^) to plot a linear regression line, the slope of which was determined as the ohmic resistance (*R*_Ohmic_). Next, we calibrated the potential responses by deducting the ohmic potential (*U* – *j*_C_·*R*_Ohmic_·*A*), and the results represent the sum of activation overpotential and equilibrium potential. We plotted a linear regression line with the corrected potential toward the current density. The slope and the *y*-intercept were used to calculate the charge transfer coefficient (α) and exchange current density (*j*_0_). Finally, we simulated the polarization curve with the values mentioned above and calculated the correlation coefficient (*R*^2^) between the model and the experimental data. In addition to the model, which incorporates the measured conductivity (σ), and assuming a homogeneous layer, the calculated ohmic resistance (*R*_Ohmic,c_) attributed by the electrolyte can be calculated using eq 10, where the compartment thickness is taken as the spacer thickness (*t* = 0.43 mm)
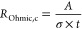
10

## 3 Results and Discussions

### 3.1 Amine Concentrations and Current Density Impact AEM System Performance

To get a better understanding of the overall performance for the AEM system with different loaded solutions, we analyzed two different operational modes (A and B) with different loaded solutions. In both sets of experiments, a CO_2_ purity higher than 99% was reached. Amines were not degraded during the experiments (no detectable concentration losses when analyzed by ion chromatography), with the longest operational windows of 7 days.

**Figure 3:**
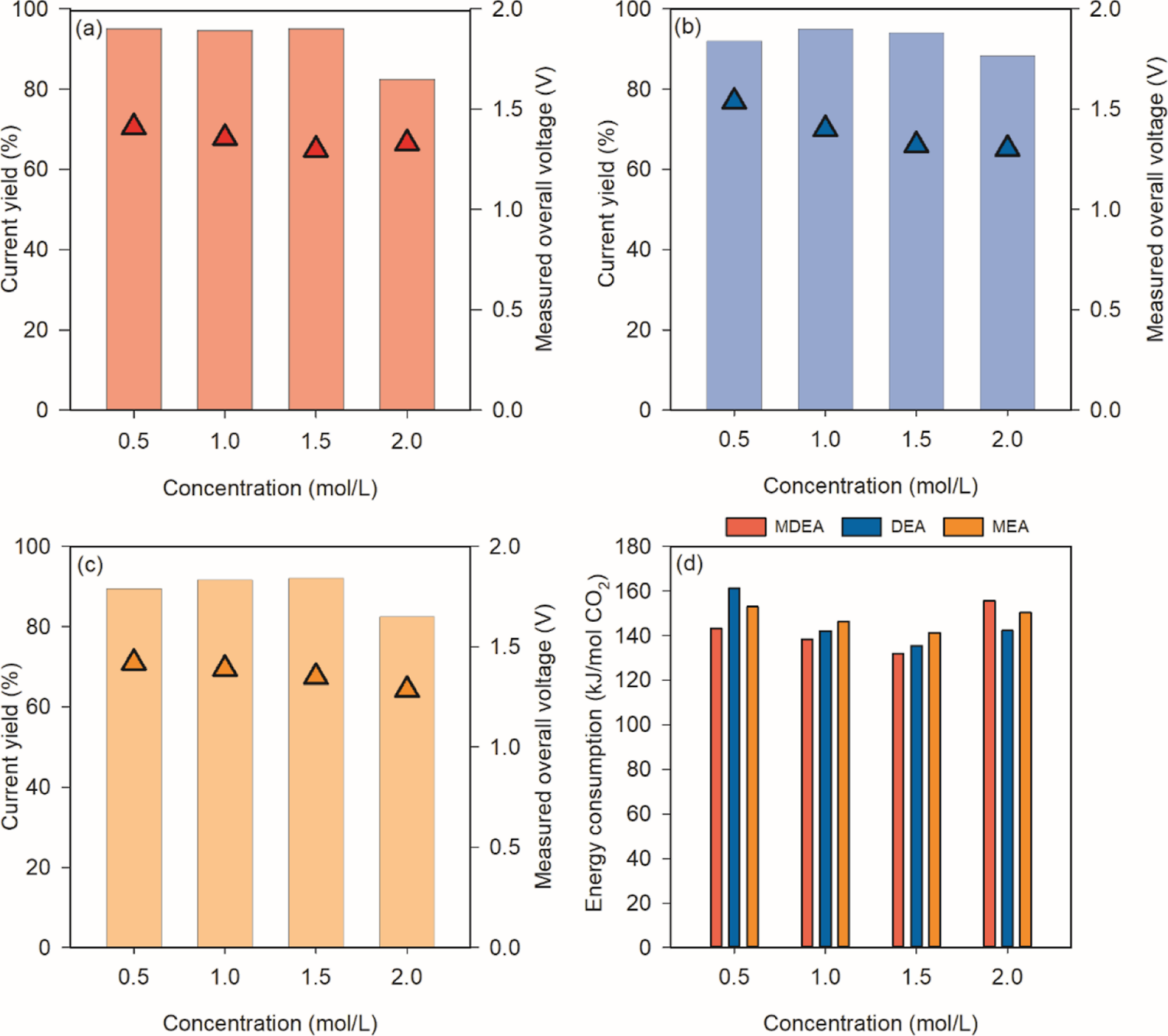
Current yield (*r*_*i*_) and measured cell voltage with increased concentration of saturated (a) MDEA, (b) DEA, and (c) MEA solution at a current density of 100 A/m^2^ under operation Mode A (bar chart: Current yield; symbols ▲: Measured overall voltage); (d) overview of the energy consumption for all cases.

The overall performance in operation mode A with different amine concentrations is shown in Figure 3. By increasing the amine concentration from 0.5 to 2 M, overall, the measured cell voltage decreases for all cases. This could be explained by the gains from the increasing conductivity (related to the decreasing *R*_Ohmic_). The current yield (*r*_*i*_) remained >95% for all cases among the groups of 0.5, 1, and 1.5 M. A decrease of *r*_*i*_ was observed when the concentration increased to 2 M. We suspected that at this high concentration, the AEM lost selectivity and did not act as an effective selective barrier and protonated amine transport occurred to a higher extent compared to the group with lower concentrations.^32^ In terms of energy consumption, a decrease was expected when we increased the concentration until a certain degree due to the increase in conductivity without loss in *r*_*i*_. However, at the highest amine concentration, there was an increase in energy consumption, most likely due to the loss of *r*_*i*_.

Among the three amines considered, MDEA demonstrated a slight advantage in terms of both the current yield and energy consumption. This advantage can be attributed to MDEA’s tertiary amine characteristics, resulting in a higher bicarbonate content in the saturated solution. In contrast, primary and secondary amines such as MEA and DEA tend to form carbamates, which contribute to a reduction in current yield. It is noteworthy that carbamate ions (R_1_NH–CO_2_^–^, R_1_R_2_N–CO_2_^–^ in our case) can permeate through the AEM to produce CO_2_, upon binding with protons. The regenerated amine (R_1_NH_2_, R_1_R_2_NH) can undergo further protonation, as illustrated in eqs 11–14. This could lead to a loss of the current yield. However, the overall differences between the amines were not significant, suggesting a high compatibility between different classes of amines and the proposed AEM process.
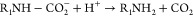
11
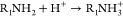
12
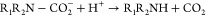
13
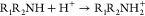
14

**Figure 4:**
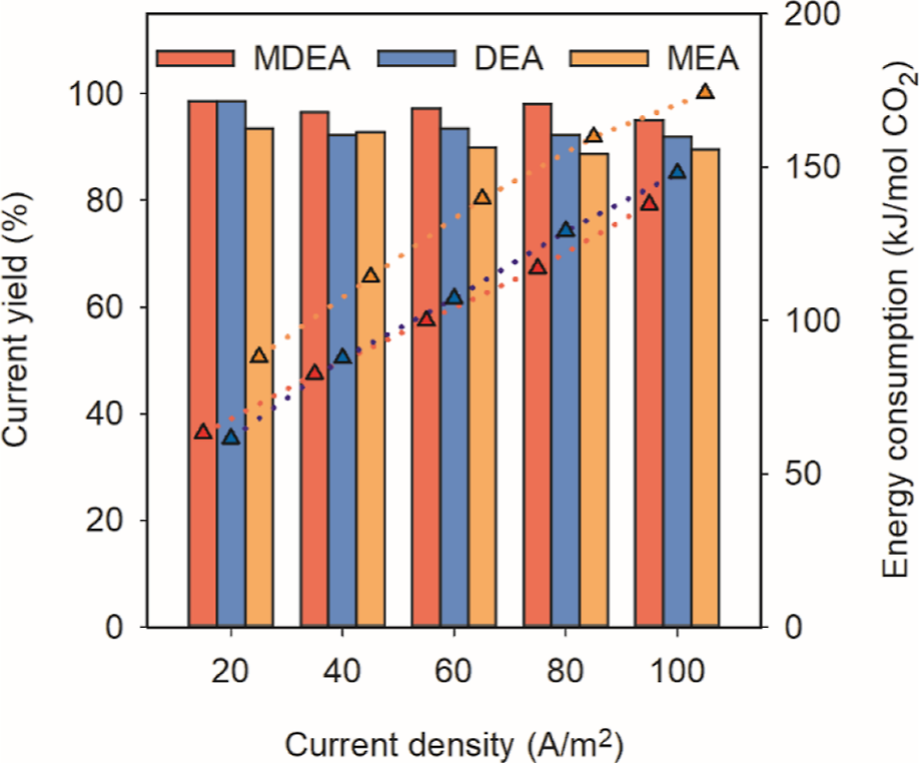
Current yield (*r*_*i*_) and energy consumption under different current densities with three different amines (0.5 M) under operation mode A (symbols -▲-: Energy consumption; bar chart: Current yield).

Figure 4 shows the overall performance of operation mode A at various current densities (0–100 A/m^2^). With all three amines at a concentration of 0.5 M, the current yield remained >90% for all cases. Particularly, we reached a current yield of 98.6% at 20 A/m^2^ for MDEA with an energy consumption as low as 63 kJ/mol. For all amines, with an increasing current density, the energy consumption for all groups increased due to the increase in applied voltage (Figure S1). The increase in the applied voltage will be further discussed in the next section. These findings highlight the efficiency of operation mode A in maintaining high current yields across various current densities and different amines.

### 3.2 MDEA Stabilizes Current Yield and Energy Consumption in a Continuous Operation

**Figure 5:**
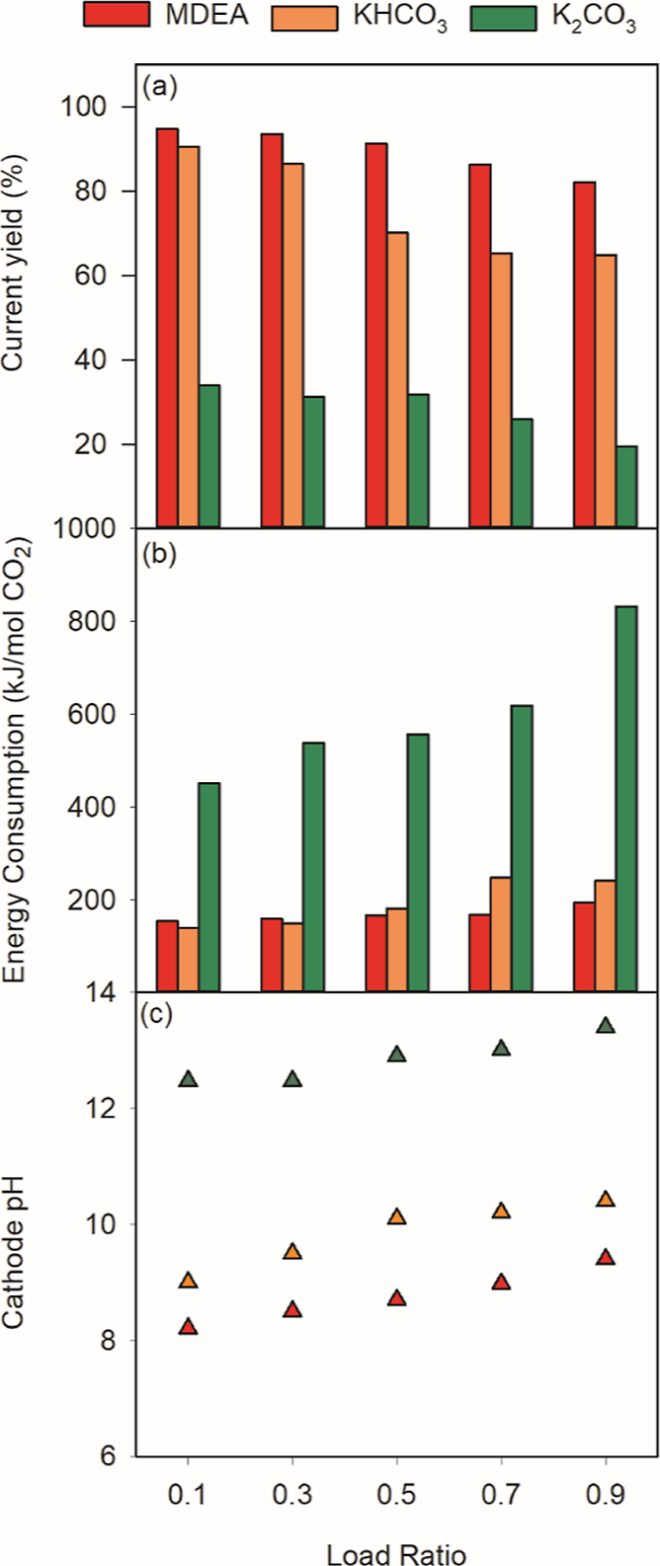
Overall performance in operation mode B with different solutions at different load ratios: (a) Current yield; (b)Energy Consumption; (c) pH of the cathode compartment at steady state.

For the study of mode B, we compared performances among 1 M saturated MDEA, 1 M KHCO_3_, and 0.5 M K_2_CO_3_ at a current density of 100 A/m^2^. As shown in Figure 5, a lower current yield (∼10%) was observed compared to that of mode A operation. Inadequate removal of CO_2_ without the vacuum and membrane contactor may explain this minor decrease: without the vacuum and the membrane contactor, the formed H_2_CO_3_ did not transform to CO_2_ fast enough, and partly diffused back to the cathodic compartment due to the concentration difference of H_2_CO_3_ between cathodic compartment and acidifying compartment.

We also observed a minor pH increase in the acidifying compartment after removing the vacuum and the membrane contactor (from 7.8 to 8.0) which indicates a higher concentration of carbon species in the steady state. MDEA had a stable performance in terms of current yield (*r*_*i*_ > 0.8 for all scenarios) across different load ratios while current yields for K_2_CO_3_ and KHCO_3_ were largely affected by the increasing load ratio, 0.91–0.64 and 0.34–0.14, respectively. The difference was due to the buffer effect, MDEA has a p*K*_a_ of 8.5 while bicarbonate/carbonate p*K*_a2_ is 10.3. With MDEA, the dominant carbon species in the cathodic compartment will remain HCO_3_^–^ [protonated MDEA (eq 15), will take up the OH^–^ instead of HCO_3_^–^ (eq 16)]. Without MDEA present, the bicarbonate will transform to carbonate when it reacts with OH^–^. Thus, the concentration of carbonate ions (CO_3_^2–^) will increase with an increasing load ratio. This could also be well verified with the pH increase from Figure 5c. The pH increased from 8.1 to 9.4 for the MDEA group, and 9.0 to 10.4 for the carbonate group. Assuming the steady state is also the chemical equilibrium state, this translates to an increase of carbonate species in the carbon system from 4.4 to 53.5% for the group of KHCO_3_, and only 0.6–10.3% for the MDEA system. As explained earlier, the transfer of 1 mol of carbonate to the acidifying compartment requires twice the current as the transfer of 1 mol of bicarbonate, for the same carbon removal. This will significantly lower the current yield, which aligns very well with the results shown in Figure 5a.
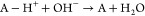
15
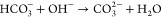
16

With the K_2_CO_3_ group, OH^–^ concentration increases with increasing load ratios in the cathodic compartment, the competition for transport between OH^–^ and carbonate species HCO_3_^–^, CO_3_^2–^ intensifies, and results in a decrease of current yield. Besides the attribution from the current yield decrease with the increasing load ratio, the increase of the overall voltage in general is another aspect that determines energy consumption (Figure S2). We suspect that the increase in cell voltage is due to the increase in membrane potential as a result of the HCO_3_^–^ concentration differences buildup across the AEM.

In Figure 5b, we obtained a better sense of how the overall energy consumption varied with different load ratios (*L*_c_). At a current density of 100 A/m^2^, the energy consumption for the MDEA group was relatively stable (154–194 kJ/mol) compared to the group of KHCO_3_ (140–262 kJ/mol), the group of K_2_CO_3_ behaves the worst among all groups, and energy consumption was both high and unstable across different load ratios (451–1148 kJ/mol).

A regeneration step is employed to reclaim a portion of dissolved CO_2_, with the regenerated solution being returned to initiate a new absorption cycle. If the recovered CO_2_ fraction is insufficient (resulting in a higher rich loading in the effluent from the regeneration process), it adversely affects the kinetics of the subsequent absorption process. Therefore, achieving a higher CO_2_ recovery rate is desirable for the overall process. In terms of energy consumption, it is noteworthy that MDEA demonstrated greater stability across a wide range of load ratios compared to that of KOH as a solution.

In practical applications, achieving full saturation of the solution with CO_2_, known as a rich loading of 100%, is rare. Instead, it is customary to anticipate a rich loading ranging from 30 to 70%, as reported in several studies.^33−36^ In general, any regeneration process is expected to produce an effluent with a lower carbon loading. As the energy consumption of our system was dependent on the carbon loading of the effluent and not on the influent, its performance can be directly correlated with different load ratios. This highlights the benefits of MDEA compared to KOH as a solution, as MDEA demonstrates stable performance characteristics over a wider range of regeneration scenarios (i.e., load ratios).

### 3.3 Buffer Effect Lowers the Cathodic Overpotential

**Figure 6:**
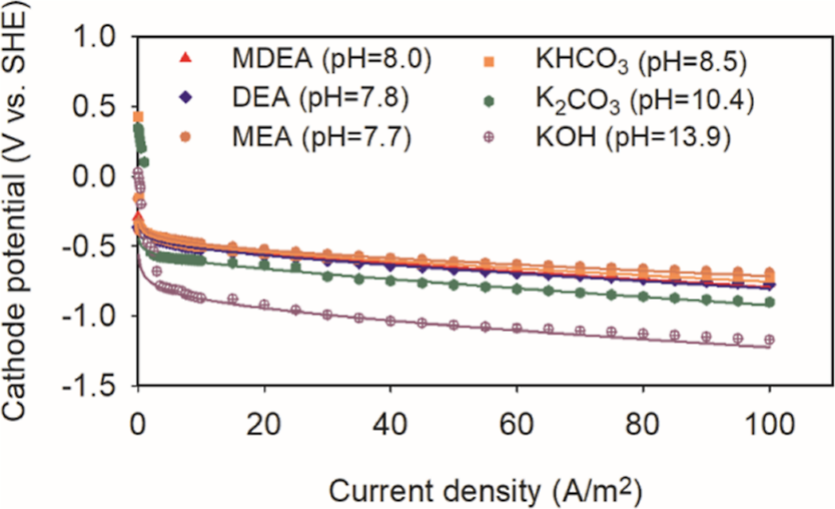
Cathode polarization curves with different solutions operating under mode A (symbols ●: experimental data; lines: model results).

The polarization curve fitting is shown in Figure 6, we noticed a displacement of the cathode potential toward more positive values relative to the pH: the groups of all three saturated amine (pH = 7.7–8) and the KHCO_3_ (pH = 8) have higher potentials than the K_2_CO_3_ group (pH = 10.4), and the KOH group (pH = 13.9) has the lowest potential among all groups. The strong correlation coefficient (*R*^2^) provided in Table 1 further confirms the robustness of this fit.

**Table 1:** Model Results for the Key Parameters and Measured Solution Parameters^a^

parameter	DEA	MEA	MDEA	K_2_CO_3_	KHCO_3_	KOH
Tafel slope a (mV/dec)^b^	–82.1	–92.1	–80.7	–70.6	–78.7	–146.5
transfer coefficient α^b^	0.72	0.64	0.73	0.84	0.75	0.40
*E*_eq_ – *b* (V)^b^	–0.58	–0.58	–0.56	–0.66	–0.56	–1.00
model *R*_Ohmic,m_ (mΩ)^b^	230.0	155.5	230.0	265.2	219.2	222.7
coefficient of determination (*R*^2^)^b^	99.6%	98.7%	99.6%	99.0%	99.4%	97.5%
measured conductivity (mS/cm)	23.7	24.2	23.8	97.4	76.4	188.8
calculated *R*_Ohmic,c_ (mΩ)	18.1	17.8	18.1	4.4	5.6	2.3
conductivity contribution	7.9%	11.4%	7.9%	1.7%	2.6%	1.0%

a The experiments were conducted under standard conditions (*T* = 298 K, *P* = 1 atm) using 1 M concentrations of KOH, KHCO_3_, saturated amine, and 0.5 M concentration of K_2_CO_3_.

b Indicates model results.

The displacement of the cathodic potential among all groups can be attributed by the difference in the sum of the equilibrium potential (*E*_eq_) and the constant part of the activation potential in the Tafel model (*b*), (*E*_eq_ – *b*) (Table 1). One way of looking at it is that buffer (either bicarbonate or protonated amine) efficiently removes the OH^–^ produced by the HER process which keeps the local pH on the cathode sufficiently low. The other way to consider such an effect is that protonated amine and bicarbonate species can directly donate their protons to produce H_2_ due to the lower binding energy with the protons compared to water molecules.^37^ The buffer effect strongly depends on the proton-binding energy of the proton-donating species, which is denoted as their p*K*_a_ (the acid dissociation constant).^21^

Despite bicarbonate and protonated amine having a similar p*K*_a_ and acting as buffers for the system, the performance of the process can differ as shown in the results of the continuous experiments (mode B). At a lower load ratio of regeneration, fully saturated KOH (KHCO_3_ group) even has a slightly better performance in terms of the energy consumption due to a lower voltage required, and this could be due to the conductivity improvement (Table 1). It is noteworthy that during the increase of the load ratio, the product concentration from the buffer reaction increases. While for the amine group, the major product is deprotonated amine, for the KHCO_3_ group, the major product is carbonate. Carbonate and hydroxyl will decrease the current yield while deprotonated amine will not. That could explain a more stable current yield and energy consumption across a wider load ratio for the MDEA group. But both (KOH and amine) suffer from a current yield loss due to the increase of the hydroxyl concentration at a higher load ratio where the buffer concentration is low.

The key parameters and measured solution parameters are summarized in Table 1. If we consider the electrolyte as a homogeneous system, with no ion concentration differences across the compartment, the Ohmic resistance calculated from the measured conductivity (*R*_Ohmic,c_) only attributed to less than 12% of the total resistance shown in the model (*R*_Ohmic,m_). The greater part of the resistance is suspected to be attributed by other factors such as bubble formation^22,38^ and conductivity decrease due to bubble formation. Bubbles, being nonconductive, can create voids within an electrolyte, increasing the local current density, also hindering the contact between the electrolyte and the electrode. A promising direction worth investing is to optimize the degassing process for both hydrogen and carbon dioxide.^22,23,38,39^ It is noteworthy that minimizing the resistance holds significance in reducing energy consumption at higher current densities, further studies should clarify internal resistance dynamics by employing electrical resistance measurements such as electrical impedance spectroscopy.^40^ Studies on PEM fuel cell and AEM fuel cell can serve as strong references refs (41 and 42). In summary, the model underscores the correlation between the enhancements facilitated by utilizing amines as the solution and the reduction in the HER potential attributed to the buffer effect. Additionally, the model reveals a relatively high calculated resistance (*R*_Ohmic_), warranting further investigation. Compared to the state-of-the-art thermal regeneration process for amine scrubbing, the use of amines and the adjustable pH swing in the electrochemical process offer the potential for increased energy efficiency. However, a critical hurdle to overcome lies in comprehending and mitigating the ohmic resistance, which is imperative for realizing the full energy-saving advantages of the electrochemical approach.

## 4 Conclusions

The proposed AEM-based electrochemical system demonstrated a very low energy demand for regenerating the loaded-alkaline amine sorbent stream used for carbon capture. The system produced CO_2_ with high purity (>99%). We successfully demonstrated an energy consumption as low as 63 kJ/mol CO_2_ at a current density (20 A/m^2^) and a high adaptability toward various amine classes. In our continuous study, MDEA showed a stable performance and superior energy efficiency compared to KOH in recovering CO_2_ under various load ratios. Our polarization curve measurements demonstrated that both amine and bicarbonate acted as effective buffers, elevating cathodic potential and consequently reducing energy consumption. Notably, amines, as employed in our system, retain the advantage over KOH solution due to the difference of their buffering properties, while protonated amines have no detrimental impact on current yield, carbonate does have detrimental impacts. We also highlighted the electrochemical cell’s resistance as an important barrier to investigate and improve for further reduction in energy consumption.

## Supporting Information Available

Cell voltage and energy consumption at different current densities with three different amines (0.5 M) under operation mode A; and cell voltage in operation mode B with different CO_2_-loaded solutions at different load ratios (PDF)
