# Supplementary material for: Energy Efficient Carbon Capture through Electrochemical pH Swing Regeneration of Amine Solution
Source: ACS Sustain Chem Eng. 2024 Apr 30;12(19):7309–17. doi: 10.1021/acssuschemeng.3c08430 (PMC11094790; doi:10.1021/acssuschemeng.3c08430)
Supplement: Supplementary file 1 — sc3c08430_si_001.pdf [file sc3c08430_si_001.pdf]

Supporting information

Energy efficient carbon capture through electrochemical pH swing regeneration of amine solution

Mu Lin [a,b], Clément Ehret [a], Hubertus V.M. Hamelers [a,b], Annemiek ter Heijne [a,b], Philipp Kuntke [a,b,*]

[a] Wetsus, European Centre of Excellence for Sustainable Water Technology, P.O. Box 1113, 8900CC Leeuwarden, The Netherlands

[b] Environmental Technology, Wageningen University, P.O. Box 17, 6700 AA Wageningen, The Netherlands

* Corresponding author e-mail: philipp.kuntke@wur.nl

Pages 2
Figure 2

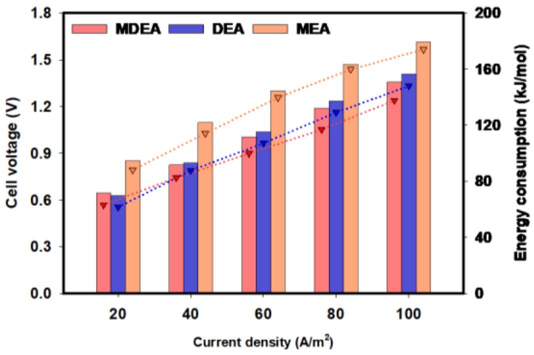

Fig. S1. Cell voltage and energy consumption at different current densities with three different amines (0.5 M) under operation mode A (Symbols -▲-: Energy consumption; bar chart: Cell voltage.)

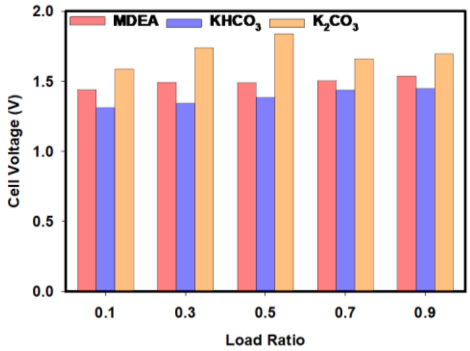

Fig. S2. Cell voltage in operation mode B with different CO_2-loaded solutions at different load ratio
